# Distraction ligamentotaxis for complex proximal interphalangeal joint fracture dislocations: a clinical study and the modified pins rubber band traction system revisited

**DOI:** 10.1186/s41038-018-0124-1

**Published:** 2018-07-23

**Authors:** Cheng Hean Lo, Simone H. Nothdurft, Hye-Sung Park, Eldho Paul, James Leong

**Affiliations:** 1 Department of Plastic and Reconstructive Surgery, Monash Health (Dandenong Hospital), 135 David St, Dandenong, Victoria 3175 Australia; 2 Monash Health (Dandenong Hospital), 135 David St, Dandenong, Victoria 3175 Australia; 3 Department of Epidemiology and Preventive Medicine, School of Public Health and Preventive Medicine, Monash University, 553 St Kilda Road, Melbourne, Victoria 3004 Australia; 4 Department of Surgery, Monash University, Wellington Road, Clayton, Victoria 3800 Australia

**Keywords:** Intra-articular fracture dislocations, Proximal interphalangeal, Pins rubber band distraction, Ligamentotaxis

## Abstract

**Background:**

The purpose of this study is to present our experience with the modified pins and rubber band traction system, discuss problems encountered, and make recommendations to optimize outcomes.

**Methods:**

Data was collected prospectively from November 2013 to March 2017 at a tertiary referral hospital in Melbourne, Australia. Patients with closed complex proximal interphalangeal joint fracture dislocations that were considered unsuitable for other surgical options were included in the study. Patients underwent dynamic skeletal distraction using the modified (Deshmukh) pins rubber band traction system. Outcomes were measured using the Nominal Rating Scale for pain; Disabilities of the Arm, Shoulder, and Hand (DASH) score; active and passive range of motion; patient rating scale; and complications.

**Results:**

Twenty patients underwent the procedure, and 19 were included in analyses. At the final follow-up assessment, an average of 62° and 77° was achieved for proximal interphalangeal joint active and passive range of motion, respectively. Pain levels were low (median score of 0 at rest and 1 ranging, out of 10). Four patients suffered minor pin site infections.

**Conclusion:**

Distraction ligamentotaxis is a useful part of the armamentarium, especially in the absence of more suitable procedures. It is important to select appropriate patients, educate, and ensure adherence to postoperative therapy. Employing the Deshmukh frame modification streamlines the theatre processes, and removal of wires at approximately 4 weeks minimizes risk of pin site infection.

## Background

Management of complex proximal interphalangeal joint (PIPJ) fracture dislocations is challenging, with the potential of long-term sequelae including pain, stiffness, and functional loss. Several treatment modalities exist, none of which consistently produce good results [1].

Early mobilization is preferred to avoid stiffness and encourage articular cartilage regeneration via restoration of synovial fluid transport [2]. Open surgical procedures may stabilize the fracture sufficiently to enable mobilization; however, this may not always be possible due to the fracture pattern. The procedure may be difficult and unforgiving; both patient selection and surgery should be approached with caution [1, 3, 4]. Open surgical procedures involve soft tissue dissection known to cause devascularization and contribute to formation of adhesions and further stiffness [3].

Dynamic joint distraction to produce ligamentotaxis is a treatment option allowing early mobilization that obviates the disadvantages inherent in an open procedure. Numerous traction devices and modifications have been introduced to restore satisfactory fracture alignment, joint congruency, and early mobilization [4–13]. Dissatisfied with the results achieved via orthotic based distraction, the authors conducted functional and biomechanical analyses and reviewed the literature [14]. Slade et al. first presented his dynamic distraction external fixation device fabricated from Kirschner (K) wires and rubber bands in 1990 at the 59th Annual Meeting of the American Society for Plastic and Reconstructive Surgery, publishing the design in 2000 [15]. Suzuki et al. [6] and Ruland et al. [16] published their experience with a dynamic skeletal system called the pins and rubber band traction system (PRTS). Various other dynamic skeletal systems have been presented in the literature [17–20]. Packham et al. [21] conducted a scoping review that comprehensively examines the applications and outcomes of these systems as well as other traction orthoses and constructs. With skeletal systems, the distraction force exerted between the hook and counter traction pins remains constant as the base for distraction is fixed, whereas any slippage or movement that occurs in orthotic based systems will result in an alteration to the distraction force [14]. Skeletal wire-based systems are light, cheap, compact, and easy to apply and achieve reasonable articular congruity without obscuring radiographic assessments [4, 5, 22]. Deshmukh et al. modified the design of the PRTS by introducing a wire frame with coils [5]. Following our recent work, Deshmukh’s modification of the PRTS became our preferred method for ligamentotaxis [14]. This modified system was first reported in the literature by Deshmukh et al. in 2004, and a small clinical series was presented [5]. Various authors have reported on the system [12, 23–26] and the benefits of the design [14, 25]. The frame can be made pre-operation potentially reducing operating times and streamlining the theatre procedures. The modified frame has coils that attach to the counter traction pin, therefore eliminating the need for this pin to rotate in the bone [5, 14, 25]. Since Deshmukh et al. solo study in 2004, the use of the design appears to have been largely forgotten. The aim of this study is to revisit Deshmukh’s frame modification and present our experience. Problems encountered and lessons learnt are discussed, and recommendations are made to avoid pitfalls and optimize patient outcomes.

## Methods

### Study population

Data was collected in a prospective clinical study conducted over more than 3 years (November 2013–March 2017) at a tertiary referral hospital in Melbourne, Australia. Potential participants with closed complex PIPJ fracture dislocations were identified in an outpatient setting. Inclusion criteria included proximal interphalangeal joint fracture dislocations that had intra-articular middle phalangeal base fractures with comminution, subluxation/dislocation, and instability. Due to complex comminution, these fractures were considered unsuitable for other surgical options such as open reduction and fixation, hemi hamate, or volar plate arthroplasty. Participants provided verbal/written consent for their treatment and inclusion in this study. Patients who were considered to be unable to adhere with the postoperative rehabilitation regime and patients with open fractures were excluded from the study. This is a clinical judgment; excluded patients may include young children (under 16 years of age), patients with cognitive impairment, tradespeople who were unable to take time off work, patients with substance addictions, and patients who openly disclosed their inability to tolerate the percutaneous pins and post-operative regime. In these cases, the patients were managed non-surgically with mobilization of the PIPJ, with or without an initial period of immobilization. The study was approved as a quality assurance activity by the Monash Health Technology & Clinical Practice Committee and Research Directorate (Project Number: 14258Q).

### Surgical technique and rehabilitation

**Fig. 1:**
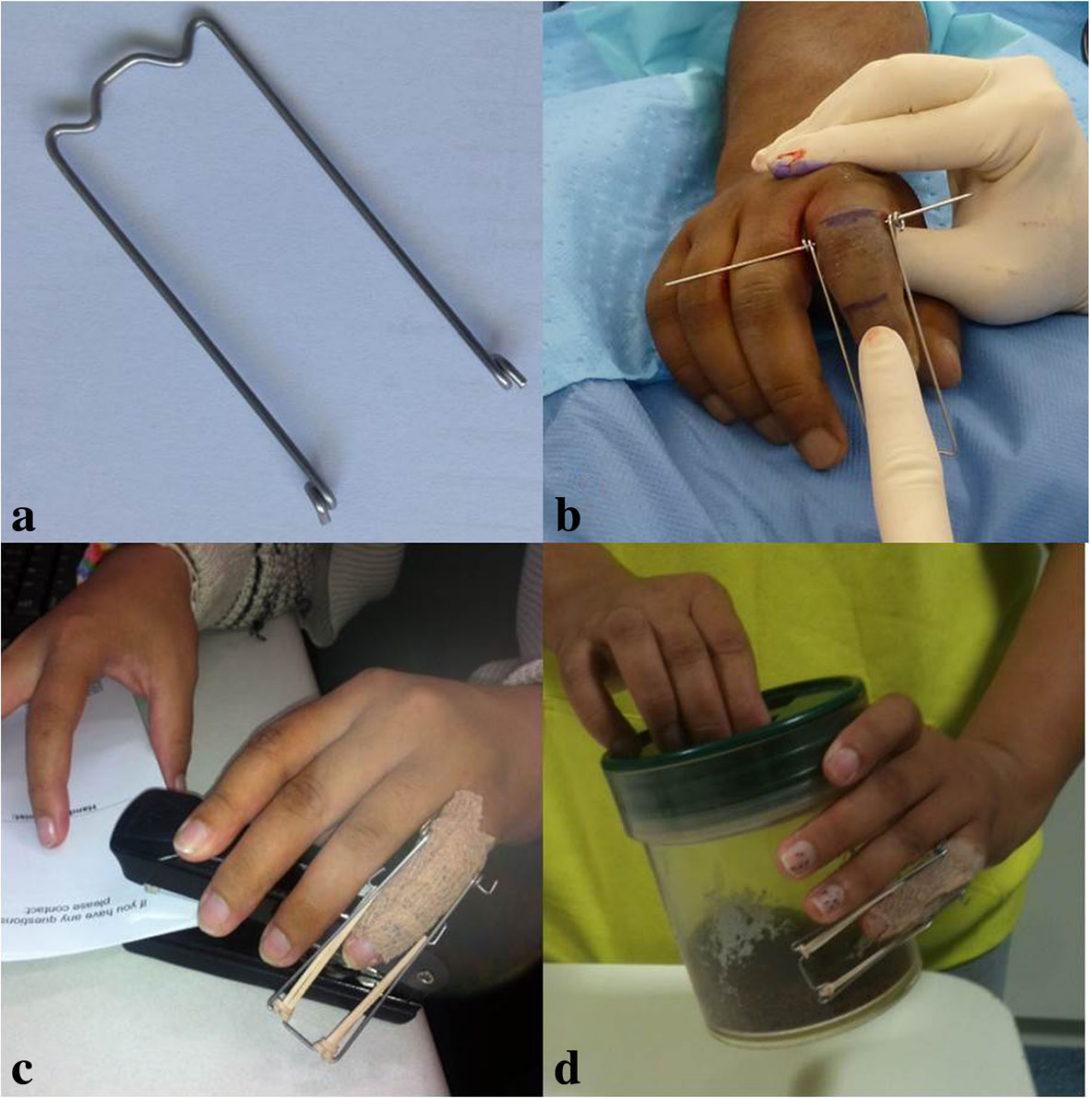
The use of distraction frames. **a** Distraction frame made and sterilized pre-operatively. **b** Application of distraction frame to counter traction pin. **c**, **d** Early return to work and activities

**Fig. 2:**
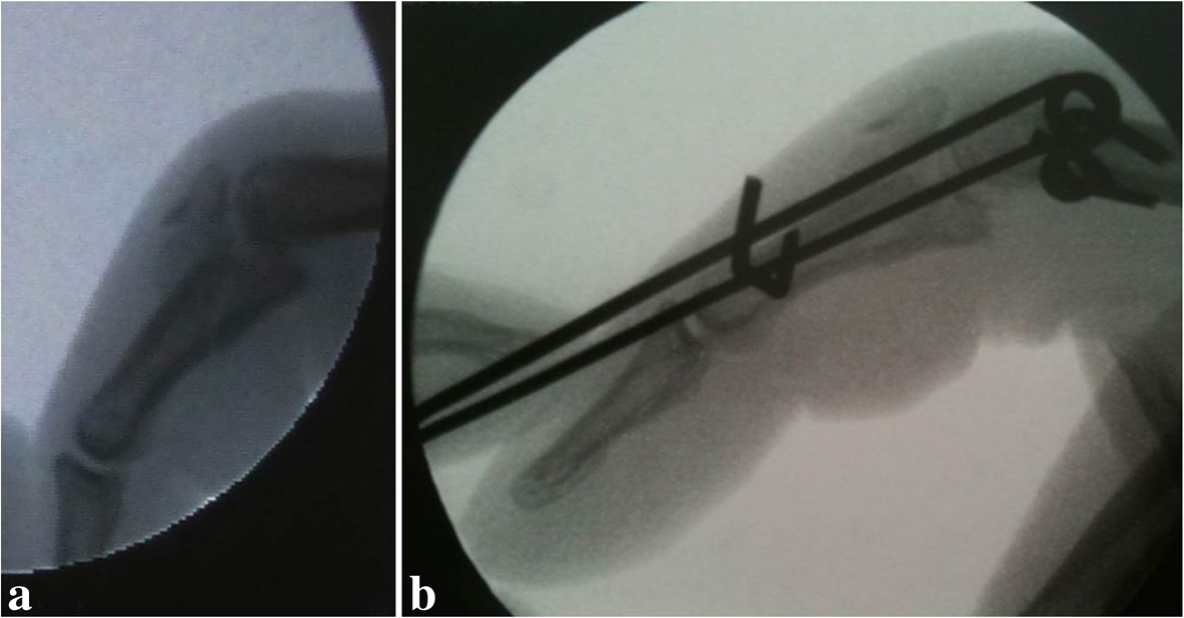
A patient with unstable index finger proximal interphalangeal joint (PIPJ) dorsal lip fracture dislocation. **a** Lateral view X-ray. **b** Intraoperative imaging to ensure fracture reduction and PIPJ space

The surgical technique used in this study was similar to that described for PRTS [5, 6]. The counter traction K-wire is passed through the head of the proximal phalanx at the level of the small elevations marking the origin of the collateral ligaments, parallel to the PIPJ line. A second K-wire (hook pin) is inserted through the distal part of the middle phalanx, parallel to the distal interphalangeal joint (DIPJ) and the counter traction pin. In this study, the distraction frames were custom made preoperatively and sterilized ready for theatre (Fig. 1a). Frames of varying sizes (small to medium approximately 2.0 × 6.5 cm; medium to large approximately 2.5 × 9.0 cm) were also pre-made and kept on shelf, then selected to suit the patient’s finger size when planning for surgery. Manufacturing instructions were available to staff to make the process efficient. The use of a pre-made frame contributed to reduce operating times by streamlining theatre procedures. The pre-made frame and sterilized single use rubber bands are applied in theatre. The tension of the rubber bands is adjusted to achieve distraction of 1 mm of the PIPJ space (Fig. 1 and 2). Joint enlocation/alignment and fracture reduction are confirmed radiologically. A third wire (reduction pin) as described by Suzuki et al. [6] is considered if there is persistent subluxation of the PIPJ. All patients received intraoperative intravenous antibiotics.

In this study, joint movement was initiated within 24–48 h of surgery. Patients were reviewed routinely at a weekly interval until removal of the K-wires at 6 weeks or when there was radiological evidence of union. X-rays were routinely performed at 1 and 6 weeks. Additional X-rays were performed on the surgeon’s request. Rubber bands were replaced weekly to prevent fatigue or breakage. As the periarticular soft tissue structures loosened over time, tension of the rubber band traction was reduced to avoid over-distraction. Differential tensioning of the rubber bands occasionally was necessary to correct minor valgus or varus angulation. Patients were educated on pin site care and elevation and given a home exercise program that incorporated mainly active and active-assisted range of motion (ROM). Whenever possible, patients returned to light functional use of the hand and work, being mindful of re-injury or over-exercising (Fig. 1c, d). Patients were reviewed at 3, 6, and 12 month intervals after surgery.

### Data and statistical analysis

Data collected include patient demographics, mechanism of injury, fracture pattern according to Kiefhaber and Stern’s classification [27], surgical management, and follow-up duration. Outcome measures collected at each review include pain (Nominal Rating Scale; 0 for no pain and 10 being the worst pain possible); Disabilities of the Arm, Shoulder, and Hand (DASH) score [28]; active and passive range of ROM; patient rating scale (Table 1); and complications. Diagnosis of pin site infection was made clinically (erythema, swelling, pain and/ or discharge). Data was summarized using mean (standard deviation (SD)) for normally distributed continuous variables, median (range) for non-normally distributed continuous variables, and number (percentage) for categorical variables. Comparisons between groups (infection vs. no infection) were made to determine potential causes or associations with wire infection using Wilcoxon rank-sum test for continuous variables and Fisher’s exact test for categorical variables. Relationships between clinical variables and functional outcomes were determined by calculating Spearman’s rank correlation coefficient (ρ). All reported *p* values are two-tailed with *p* < 0.05 indicating statistical significance. Data were collected in Microsoft Excel® for Mac 2011 (Version 14.6.6) and statistical analyses performed using SAS version 9.4 (© SAS Institute Inc).

**Table 1:** Patient survey—satisfaction with hand appearance, progress and abilities to complete light, and heavy activities

Patient satisfaction	Score				
	1	2	3	4	5
Hand appearance	Extremely happy	Moderately happy	Neutral	Moderately unhappy	Extremely unhappy
Progress	Extremely happy	Moderately happy	Neutral	Moderately unhappy	Extremely unhappy
†Light activities	Able to complete all tasks	Able to complete 75% of tasks	Able to complete 50% of tasks	Able to complete 25% of tasks	Unable to complete any tasks
† †Heavy activities	Able to complete all tasks	Able to complete 75% of tasks	Able to complete 50% of tasks	Able to complete 25% of tasks	Unable to complete any tasks

†Light activities—any activity not requiring a weight greater than 1 kg, or a strong gripping action

† †Heavy activities—any activity requiring a weight greater than 1 kg or a strong gripping action such as opening a jar or holding a hammer

## Results

### Patient demographics

Twenty patients underwent dynamic distraction ligamentotaxis using the modified PRTS for a complex PIPJ fracture (Table 2). In ten cases, the affected hand was the dominant hand. The first author managed 12 patients; a second surgeon managed three patients, and five other surgeons treated one patient each. The median time from injury to surgery was 10 days (range 1–28). In one case, an additional dorsal blocking K-wire was inserted to control the dorsal fragment in a pilon-type injury. None of the patients had a third “reduction pin” inserted as described by Suzuki et al. [6]. All fractures reduced satisfactorily following intra-operative application of the distraction frame and the rubber band adjustments to achieve the desired joint space (Fig. 2). Patients underwent a median of 41 days (range 23–66) of distraction.

**Table 2:** Demographics of patients undergoing distraction ligamentotaxis

Demographic	Result
Total number of patients	20 patients
Gender	Male = 15
	Female = 5
Age	Range = 21–62 years
	Mean (SD) = 37.6 (9.9) years
Etiology	Sporting activities = 10
	Other accidents = 8
	Altercation = 2
Hand dominance	Right = 19
	Left = 1
Injured hand	Right = 11
	Left = 9
Injured digit	Little = 10
	Ring = 5
	Middle = 4
	Index = 1
Keifhaber Classification [27]	Pilon = 9
	Palmar lip, unstable = 6
	Dorsal lip, unstable = 4
	Palmar lip, tenuous = 1

From the total of 20 patients, one patient was lost to follow-up, after unexpectedly returning home overseas soon after surgery. The remaining 19 patients were followed up for a median of 33 weeks (range 4–119), and their outcomes analyzed. Nine patients did not attend their follow-up appointment within the first year post distraction despite repeated reminders. Several of these patients lived over 2 h away from the hospital. One patient did not attend due to incarceration. Two patients proceeded to pursue treatment elsewhere 22 weeks following distraction, and 5 months prior to the end of the study period respectively. One patient found the distraction process overwhelming requiring psychological counseling. This patient did not attend several of the follow-up appointments.

### Pain and function

The reported Nominal Rating Scale for pain (0–10) remained low throughout the postoperative period. At the last visit, the median pain score was 0 at rest and 1 with joint ranging. Two patients complained of pain affecting mobilization during distraction. At the final follow-up assessment, an average of 62° (SD 22°) and 77° (SD 15°) were achieved for active and passive PIPJ flexion ROM respectively. Active and passive DIPJ flexion ROM were 54° and 66° respectively.

The median DASH score achieved at the final assessment was 6.7 (range 0–55). Patients were able to complete almost all light and heavy activities, reporting median scores of 3 (hand appearance), 2 (satisfaction with progress), 1 (light activities), and 2 (heavy activities) (Table 1).

### Infection

Four patients were diagnosed clinically with pin site infections (one patient with *Staphylococcus aureus*; one patient with Methicillin-resistant *Staphylococcus aureus*; two patients did not have microbiological investigations). All four patients were initially prescribed empirical cephalosporin covering Gram-positive cocci, the antibiotic of choice later tailored according to microbiological findings in two cases. In all cases, the K-wires were removed as soon as the diagnosis of infection was made, i.e., at 23, 32, 33, and 42 days after insertion. In three cases, the removal of the K-wires was earlier than planned. There was a significant association between pin site infection and the finger affected, as all four cases of infection occurred in a little finger (*p* = 0.03). A significant association was not demonstrated between duration of distraction frame application and infection, with a median of 33 days (range 23–42) in the infected group as opposed to a median of 41 days (range 31–66) in the non-infected group (*p* = 0.09). No significant associations were found between infection and patient age, gender, hand dominance, fracture pattern, or time from injury to surgery.

### Range of motion

Using Spearman’s rank correlation coefficient (*ρ*), no correlation was found between length of time from injury to surgery and active PIPJ ROM achieved (*ρ* = − 0.004, *p* = 0.99). There was no significant correlation between duration of follow-up and outcome measures including active PIPJ ROM achieved (*ρ* = 0.22, *p* = 0.36).

## Discussion

Complex PIPJ fracture dislocations are uncommon. Twenty cases were recruited for distraction ligamentotaxis using the modified PRTS during the study. Almost all patients had severe pilon-type fractures or fractures with instability (Table 2). The surgical technique was considered to be easy to learn and reproducible and potentially could be performed proficiently by surgeons with a range of experience. The pre-made frame assisted to streamline the surgical procedure and was made efficiently before the operation. A third K-wire (reduction pin) was not necessary in most cases. Distraction ligamentotaxis was a useful part of the armamentarium, especially in the absence of more suitable procedures.

**Fig. 3:**
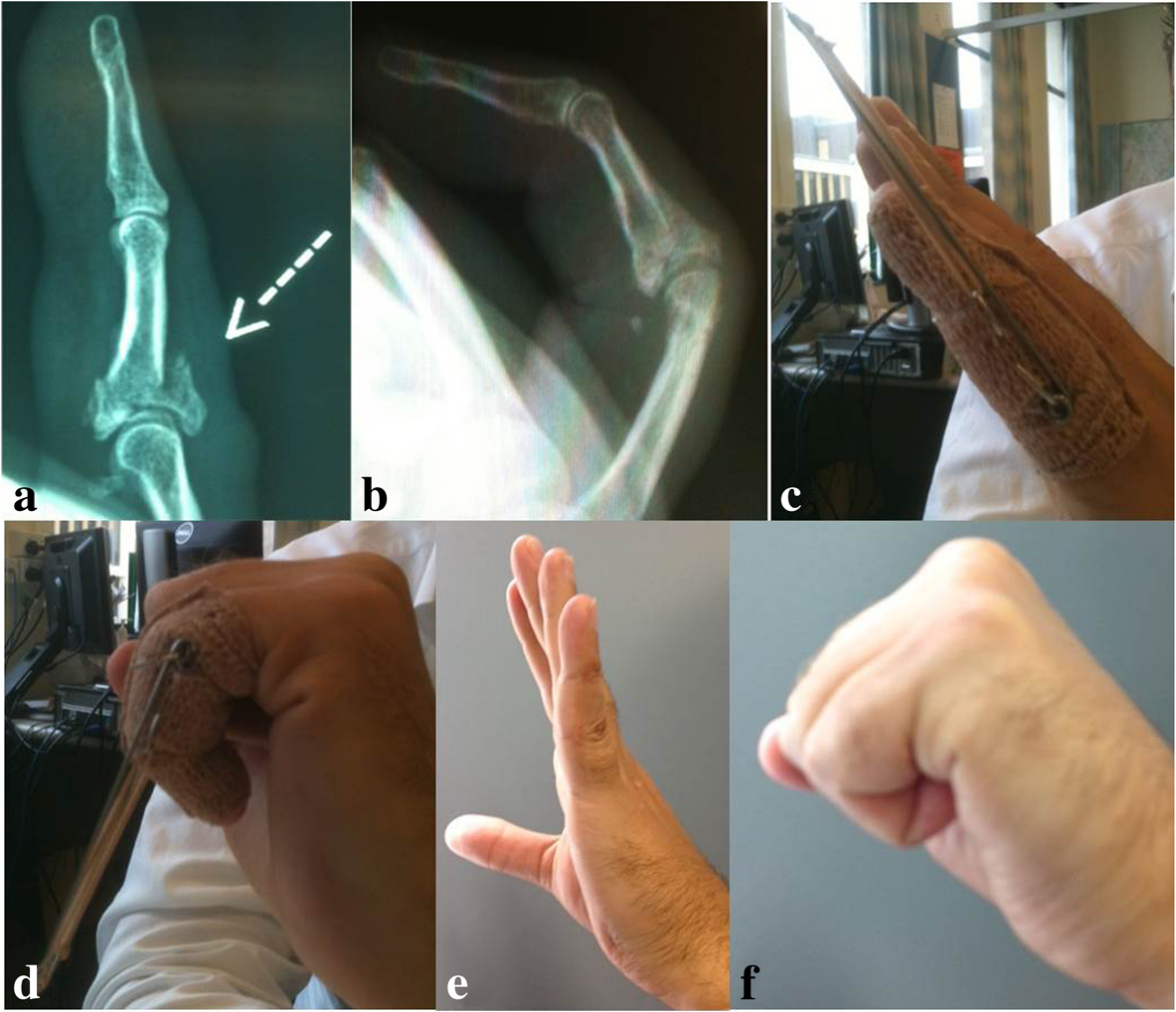
A patient with left little finger pilon type proximal interphalangeal joint (PIPJ) fracture. **a** Pre-operative X-ray. **b** X-ray post removal of distraction frame demonstrating satisfactory fracture reduction and healing. **c**, **d** Early active motion with distraction frame. **e**, **f** Excellent range of motion (ROM) achieved

The average active and passive PIPJ ROM achieved in this series of 62° and 77°, respectively, were relatively low compared to published literature (Table 3). In their literature reviews, Deshmukh et al. [5] reported a range of 76°–101° and Debus et al. [23] reported an average active PIPJ ROM of 78° (range 64°–95°). Albeit disappointing, this may be attributed to a variety of factors. Firstly, in this study, distraction ligamentotaxis was reserved for complex fractures (with comminution, instability, delayed presentation) or fractures considered unsuitable for open surgical procedures (Fig. 3). As patient selection and inclusion criteria for distraction ligamentotaxis is not standardized in the literature, interpretations and comparisons with other published series should be carried out in perspective. In this series, other surgical options often did not exist nor were superior results a certainty with other conservative options. Secondly, many of our patients did not attend their hand therapy appointments. The PIPJ ROM achieved may be a reflection of this poor adherence to therapy. Encouragingly, delayed patient presentation or delayed surgery did not lead to poorer PIPJ ROM.

**Table 3:** Summary of comparable published series

Author	Distraction technique	Number of patients	Mean active PIPJ ROM (°)	Infection rate (%)
Suzuki et al. [6]	Suzuki PRTS	5	80	unknown
De Soras et al. [4]	Suzuki PRTS	11	84	9
De Smet & Fabry [22]	Suzuki PRTS	5	63	20
Majumber et al. [29]	Suzuki PRTS	14	74	21
Duteille et al. [11]	Suzuki PRTS	20	86	5
Deshmukh et al. [5]	Deshmukh PRTS	13	85	15
Keramidas et al. [1]	Suzuki PRTS	11	84	18
Ellis et al. [12]	Slade PRTS	8	88	13
Agarwal et al. [24]	Suzuki PRTS	25	67†	28
Ruland et al. [16]	Slade PRTS	34	88†	24
Kneser et al. [30]	Suzuki PRTS	5	74	20
Debus et al. [23]	Suzuki PRTS	15	66	20
Finsen [25]	Suzuki PRTS	18	72	17
Current series	Deshmukh PRTS	20	62	21

*PIPJ* proximal interphalangeal joint, *PRTS* pin and rubber band traction system, *ROM* range of motion

†Not specified in publication if measured ROM was active or passive

The pin site infection rate was well within the reported range (5–28%) in published series involving PRTS and modified PRTS (Table 3) [11, 24]. In the series of 13 patients, Deshmukh et al. [5] had two patients (15 %) with minor pin track infections (treated with oral antibiotics but not removal of wire), comparing favorably with most published series involving Suzuki’s original description of PRTS [6]. Deshmukh et al.’s modified PRTS [5] has the advantages of the original Suzuki method (1994) but with the additional benefit of avoiding wire rotation and friction at the bone-pin interface, an accepted cause of pin track infection and bone suppuration [23, 25]. A lower infection rate could not be demonstrated in the current series; however, this was thought to be influenced by patient psychosocial factors. In one patient, non-adherence to treatment was a major contributing factor (drug use, absconded from inpatient care, did not attend appointments). This case highlights the importance of appropriate patient selection and education. Inability or unwillingness to adhere to postoperative therapy or attend appointments should be considered relative contraindications to surgery. In a second patient, the diagnosis of pin site infection was potentially over inclusive, based on mild pin site erythema and irritation only. The symptoms resolved rapidly with careful pin site care and prophylactic oral antibiotics. In most cases, successful management of pin site infection involved oral antibiotics, with or without premature removal of infected K-wires. Agarwal et al. (2007) proposed that the width of frame must be appropriately wide to avoid erosion into skin yet sufficiently narrow to avoid being cumbersome and impedance on adjacent digits [24].

In published literature, there is no consensus regarding optimal duration of distraction with a range of 2–7 weeks being recommended [5, 6, 24, 29]. In our series, we removed the K-wires at 6 weeks or at radiological evidence of union, generally maintaining distraction longer due to the complex nature of our patient’s fractures (including comminution and depressed articular segments). Due to the size of our clinical series and lack of statistical power, a significant relationship between duration of distraction and pin site infection could not be demonstrated. However, in the subgroup of four patients who did develop pin site infections, three patients underwent distraction for more than 4 weeks. In the one case where pin site infection developed relatively early at 23 days, the patients’ non-adherence to treatment was a major contributing factor. We believe that prolonged distraction does carry a risk of pin site infection, and our preference now is to remove K-wires at 4 weeks whenever possible.

There was a statistically significant association between infection and the little finger (*p* = 0.03), as all four cases of infection occurred in the little finger. Being a border digit, the little finger with applied distraction frame is more likely knocked and subject to trauma, leading to wire rotation and friction and hence infection.

In our series, pain levels remained low after surgery and gradually improved over time. Patients were able to complete almost all tasks and were overall happy with hand appearance and satisfied with progress (Table 1). Our median DASH score of 6.7 compared favorably to the published literature [30]. Reduced DIPJ range of motion has been reported by several authors, attributed to transfixion of the lateral bands and potentially preventable by vigilant postoperative therapy [5, 6, 23–25]. The active and passive DIPJ flexion ROM achieved in our current series of 54° and 66°, respectively, also compared favorably.

The surgical unit involved is part of a large tertiary referral center with more than 25 surgeons, including a subgroup of surgeons who perform hand surgery on a regular basis. The main author managed 12 patients, and six other surgeons managed eight patients. The general consensus was that surgeon heterogeneity did not impact the results. The technique, simplified by using a pre-made frame, was considered to be a technique that could be readily implemented in any unit with interested surgical and therapy personnel. This series involved a single group of patients selected and treated with the modified PRTS. As a prospective cohort study, there was no randomization and no control group, and this may be considered a limitation in the study. The patients selected for dynamic ligamentotaxis, however, were considered unsuitable for other management options; consequently, comparisons with other patient groups are difficult, such as those treated non-surgically or patients who underwent open surgical procedures. In addition, comparisons with other published series involving PRTS should be performed with care.

## Conclusion

The modified PRTS was first used in a clinical study and reported in the literature by Deshmuhk et al. in 2004. Until now, this was a solo publication, and despite other authors referring to the system and its benefits, the design had been largely forgotten. This clinical study revisited the system and the results have been outlined. Pitfalls and benefits have been reported. The authors believe that the modified PRTS produces results comparable to other studies and it should be considered in the management of complex proximal interphalangeal joint fracture dislocations. To optimize outcomes, the authors recommend careful patient selection; the meticulous care of the pin sites to avoid infection; and consideration of an earlier removal of frames if sufficient bone healing and ligamentotaxis has occurred. Most importantly, a commitment from the surgeon and the hand therapist is essential to educate and review the patients on a regular basis, avoiding the outsourcing of care.

## Abbreviations

DASH: Disabilities of the Arm, Shoulder, and Hand
DIPJ: Distal interphalangeal joint
K: Kirschner
PIPJ: Proximal interphalangeal joint
PRTS: Pins rubber band traction system
ROM: Range of motion
SD: Standard deviation

## References

[1] Keramidas E, Solomos M, Page RE, Miller G (2007). The Suzuki frame for complex intra-articular fractures of the proximal interphalangeal joint of the fingers. Ann Plast Surg.

[2] Salter RB, Simmonds DF, Malcolm BW, Rumble EJ, MacMichael D, Clements ND (1980). The biological effect of continuous passive motion on the healing of full-thickness defects in articular cartilage. An experimental investigation in the rabbit. J Bone Joint Surg Am.

[3] Stern PJ, Roman RJ, Kiefhaber TR, McDonough JJ (1991). Pilon fractures of the proximal interphalangeal joint. J Hand Surg Am..

[4] De Soras X, De Mourgues P, Guinard D, Moutet F (1997). Pins and rubbers traction system. J Hand Surg Br..

[5] Deshmukh SC, Kumar D, Mathur K, Thomas B (2004). Complex fracture-dislocation of the proximal interphalangeal joint of the hand. Results of a modified pins and rubbers traction system. J Bone Joint Surg Br.

[6] Suzuki Y, Matsunaga T, Sato S, Yokoi T (1994). The pins and rubbers traction system for treatment of comminuted intraarticular fractures and fracture-dislocations in the hand. J Hand Surg Br..

[7] Allison DM (1996). Fractures of the base of the middle phalanx treated by a dynamic external fixation device. J Hand Surg Br.

[8] Schenk RR. Dynamic traction and early passive movement for fractures of the proximal interphalangeal joint. J Hand Surg Am. 1986;11(6):850–8. 10.1016/s0363-5023(86)80236-2 3794242

[9] Inanami H, Ninomiya S, Okutsu I, Tarui T (1993). Dynamic external finger fixator for fracture dislocation of the proximal interphalangeal joint. J Hand Surg Am..

[10] Hynes MC, Giddins GE (2001). Dynamic external fixation for pilon fractures of the interphalangeal joints. J Hand Surg Br..

[11] Duteille F, Pasquier P, Lim A, Dautel G (2003). Treatment of complex interphalangeal joint fractures with dynamic external traction: a series of 20 cases. Plast Reconstr Surg.

[12] Ellis SJ, Cheng R, Prokopis P, Chetboun A, Wolfe SW, Athanasian EA (2007). Treatment of proximal interphalangeal dorsal fracture-dislocation injuries with dynamic external fixation: a pins and rubber band system. J Hand Surg Am.

[13] Abou Elatta MM, Assal F, Basheer HM, El Morshidy AF, Elglaind SM, Abdalla MA (2017). The use of dynamic external fixation in the treatment of dorsal fracture subluxations and pilon fractures of finger proximal interphalangeal joints. J Hand Surg Eur.

[14] Nothdurft SH, Hartman J, Park HS, Lo CH. Dynamic distraction of the PIPJ: the importance of the proximal K-wire. In: Proceedings of the Australian Hand Therapy Association Annual Conference. Glenelg; Australian Hand Therapy Association. 2017. p. 30. http://ahtaconference.com.au/#2017-presentations.

[15] Slade JF, Baxamusa TH, Wolfe SW (2000). External fixation of proximal interphalangeal joint fracture-dislocations. Atlas Hand Clin.

[16] Ruland RT, Hogan CJ, Cannon DL, Slade JF (2008). Use of dynamic distraction external fixation for unstable fracture-dislocations of the proximal interphalangeal joint. J Hand Surg Am..

[17] Fahmy NR (1990). The Stockholm serpentine spring system. J Hand Surg Br..

[18] Gaul JS, Rosenberg SN (1998). Fracture-dislocation of the middle phalanx at the proximal interphalangeal joint: repair with a simple intradigital traction-fixation device. Am J Orthop (Belle Mead NJ).

[19] Krakauer JD Stern PJ (1996). Hinged device for fractures involving the proximal interphalangeal joint. Clin Orthop Relat Res.

[20] Korting O, Facca S, Diaconu M, Liverneaux P (2009). Treatment of complex proximal interphalangeal joint fractures using a new dynamic external fixator: 15 cases. Chir Main.

[21] Packham TL, Ball PD, MacDermid JC, Bain JR, DalCin A (2016). A scoping review of applications and outcomes for the management of intra-articular fractures and fracture dislocations in the hand. J Hand Ther.

[22] De Smet L, Fabry G (1998). Treatment of fracture-dislocations of the proximal interphalangeal joint with the “pins & rubbers” traction system. Acta Orthop Belg.

[23] Debus G, Courvoisier A, Wimsey S, Pradel P, Moutet F (2010). Pins and rubber traction system for intra-articular proximal interphalangeal joint fractures revisited. J Hand Surg Eur..

[24] Agarwal AK, Karri V, Pickford MA (2007). Avoiding pitfalls of the pins and rubbers traction technique for fractures of the proximal interphalangeal joint. Ann Plast Surg.

[25] Finsen V (2010). Suzuki’s pins and rubber traction for fractures of the base of the middle phalanx. J Plast Surg Hand Surg.

[26] Shen XF, Mi JY, Rui MY, Chou J, Tian J, Chim H. Delayed treatment of unstable proximal interphalangeal joint fracture-dislocations with a dynamic external fixator. Injury. 2015;46(10):1938–44. 10.1016/j.injury.2015.06.027 26144906

[27] Kiefhaber TR, Stern PJ (1998). Fracture dislocations of the proximal interphalangeal joint. J Hand Surg Am..

[28] Hudak PL, Amadio PC, Bombardier C (1996). Development of an upper extremity outcome measure: the DASH (disabilities of the arm, shoulder and hand) [corrected]. The Upper Extremity Collaborative Group (UECG). Am J Ind Med.

[29] Majumder S, Peck F, Watson JS, Lees VC (2003). Lessons learned from the management of complex intra-articular fractures at the base of the middle phalanges of fingers. J Hand Surg Br..

[30] Kneser U, Goldberg E, Polykandriotis E, Loos B, Unglaub F, Bach A (2009). Biomechanical and functional analysis of the pins and rubbers tractions system for treatment of proximal interphalangeal joint fracture dislocations. Arch Orthop Trauma Surg.

